# Inside/Outside: Post‐Synthetic Modification of the Zr‐Benzophenonedicarboxylate Metal–Organic Framework

**DOI:** 10.1002/chem.201903630

**Published:** 2020-02-04

**Authors:** Alexander Mohmeyer, Malte Schäfer, Andreas Schaate, Sonja Locmelis, Andreas M. Schneider, Peter Behrens

**Affiliations:** ^1^ Institute for Inorganic Chemistry Leibniz University Hannover Callinstr. 9 30167 Hannover Germany; ^2^ Cluster of Excellence PhoenixD, (Photonics, Optics, and Engineering—Innovation Across Disciplines) Hannover Germany

**Keywords:** benzophenone, modulation, photochemistry, post-synthetic modification, zirconium

## Abstract

The Zr‐based metal–organic framework, Zr‐*bzpdc*‐MOF, contains the photoreactive linker molecule benzophenone‐4,4′‐dicarboxylate (*bzpdc*) which imparts the possibility for photochemical post‐synthetic modification. Upon irradiation with UV light, the keto group of the benzophenone moiety will react with nearly every C−H bond‐containing molecule. Within this paper, we further explore the photochemical reactivity of the Zr‐*bzpdc*‐MOF, especially with regard to which restrictions govern internal versus external reactions. We show that apart from reactions with C−H bond‐containing molecules, the MOF reacts also with water. By studying the reactivity versus linear alcohols we find a clear delineation in that shorter alcohol molecules (up to butanol as a borderline case) react with photoexcited keto groups throughout the whole crystals whereas longer ones react only with surface‐standing keto groups. In addition, we show that with the alkanes *n*‐butane to *n*‐octane, the reaction is restricted to the outer surface. We hypothesize that the reactivity of the Zr‐*bzpdc*‐MOF versus different reagents depends on the accessibility of the pore system which in turn depends mainly on the size of the reagents and on their polarity. The possibility to direct the post‐synthetic modification of the Zr‐*bzpdc*‐MOF (selective modification of the whole pore system versus surface modification) gives additional degrees of freedom in the design of this metal–organic framework for shaping and for applications.

## Graphical abstract

**Inside/Outside**: The photochemical post‐synthetic modification of the Zr‐benzophenone‐dicarboxylate‐MOF can be directed to occur only on the surface or throughout the whole crystals, providing additional possibilities to selectively introduce novel functionalities and tailor the properties of this MOF. Major control parameters are size and polarity of the modifying reagent.

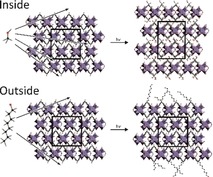

## Introduction

Metal‐organic frameworks (MOFs) feature an ordered structure with permanent porosity which is of high interest for a variety of applications like catalysis,^1^ gas storage and separation,^2^, ^3^ sensing,^4^, ^5^, ^6^ energy storage and conversion^7^ or biomedicine.^8^, ^9^ To fully exploit this potential, the structure and the properties of a MOF need to be fine‐tuned to the specific intended application in order to achieve optimum results. MOFs are well prepared to meet such challenges, as their modular construction from metal oxide nodes and organic linker molecules allows the introduction of various properties by deliberately choosing the metal and the linker molecule. However, certain specific combinations of properties are difficult to elaborate due to the rather harsh synthesis conditions employed. Especially, some desired functionalities at the organic linker molecules may be too sensitive to withstand the synthesis environment. Therefore, other strategies have been developed to overcome such restrictions imposed by the use of labile or more sophisticated functional groups at the linker molecules during synthesis. One of the most strongly investigated approach to modify and functionalise MOFs is the post‐synthetic modification (PSM) of the framework. Several routes like linker exchange, post‐synthetic metalation or organic reactions at the linker molecules have been developed.^10^, ^11^, ^12^, ^13^

Especially, the versatility of organic reactions to covalently modify the linker molecules has been widely studied and utilised to introduce different functionalities to the framework after the MOF skeleton has been formed. Such PSM reactions are usually performed on specific functionalities of the organic linker molecules like amino, alkyne, azide or halide groups or by click chemistry. A major requirement to ensure the success of such post‐synthetic treatments is the stability of the framework during the PSM.^10^, ^11^, ^14^, ^15^, ^16^ MOFs with Zr^IV^‐based nodes are especially appropriate for PSM due to their high stability towards thermal and chemical stress,^17^, ^18^ making them ideal platforms for introducing different functionalities by chemical transformations and modifications with the aim to adjust the properties of the material with a specific application in mind.^13^, ^16^, ^19^, ^20^, ^21^

**Scheme 1:**
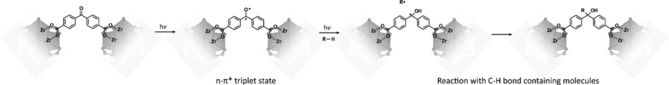
Schematic mechanism for the photoreaction of the keto group of benzophenone units in the framework of Zr‐*bzpdc*‐MOF: irradiation leads to the excitation of the keto group resulting in a biradicaloid triplet state which then reacts with a C−H bond‐containing molecule, resulting in the formation of a C−C bond and the reduction of the keto group.^23^

Recently, we have introduced a novel type of PSM reaction. This makes use of the specific reactivity of the benzophenone moiety. Upon excitation by irradiation with UV light, the ketyl radicals formed react with practically any C−H bond of an organic molecule according to the reaction mechanism schematically shown in Scheme 1.^22^, ^23^ We have employed the linker molecule benzophenone‐4,4′‐dicarboxylic acid (H_2_ *bzpdc*) to synthesize the Zr‐based metal–organic framework Zr‐*bzpdc*‐MOF.^24^ The synthesis made use of the modulation method^25^, ^26^ and lead to a compound with formula Zr_6_O_4_(OH)_6_(HCO_2_)_2_(*bzpdc*)_4_. Stock and co‐workers have used this linker for the construction of the MOF CAU‐8 with the formula Al(OH)(O_2_C‐C_6_H_4_‐CO‐C_6_H_4_‐CO_2_) and have studied its photochemical behavior.^27^, ^28^ The Zr‐*bzpdc*‐MOF has a two‐dimensional layered structure, can be delaminated to thin nanosheets and exhibits a moderate porosity of 650 m^2^ g^−1^. Its high chemical and thermal stability make it highly suitable for PSM reactions. In a preliminary study, we have converted this MOF in photochemical PSM reactions with decane and PEG and have observed drastically different wetting properties of the products.^24^ Furthermore, we have initiated photochemical grafting‐from polymerisation reactions of EDOT (3,4‐ethylenedioxythiophene) to yield nanoporous electrically conducting MOF‐PEDOT composites.^29^ In all cases, the powder X‐ray diffraction pattern of the material remained unchanged; accordingly, the modification had only taken place on the surface of the MOF crystals. The central question which then arises is whether the photochemical PSM is in general only possible at the outer surface or whether some characteristics of the organic molecule, like its size or its polarity, decide about whether the reaction takes place only at the surface or throughout the Zr‐*bzpdc*‐MOF crystal.

In the present study, we systemically investigate the photochemical PSM of the Zr‐*bzpdc*‐MOF. First, we compare the reactivity and the obtained products of the reactions of the linker molecule H_2_ *bzpdc*, reacted either as a free molecule or when immobilized in the framework. This part focusses on the reactions with simple molecules, namely methanol and ethanol as well as water. Only few studies have reported on the reactions of benzophenone in aqueous media;^30^, ^31^, ^32^, ^33^ in the present case, these may be especially important, as in the laboratory the MOF samples are exposed to light and air humidity when no special precautions are taken. In the second part of the study, we have reacted the Zr‐*bzpdc*‐MOF with series of alkanes and alcohols of different chain length. We show that the PSM takes place either throughout the MOF crystals or only on the surface, depending on the polarity and on the chain lengths of the probe molecules. These results allow to design PSM strategies of this photoreactive MOF in view of different applications.

## Results and Discussion

### Synthesis of Zr‐*bzpdc*‐MOF

**Figure 1:**
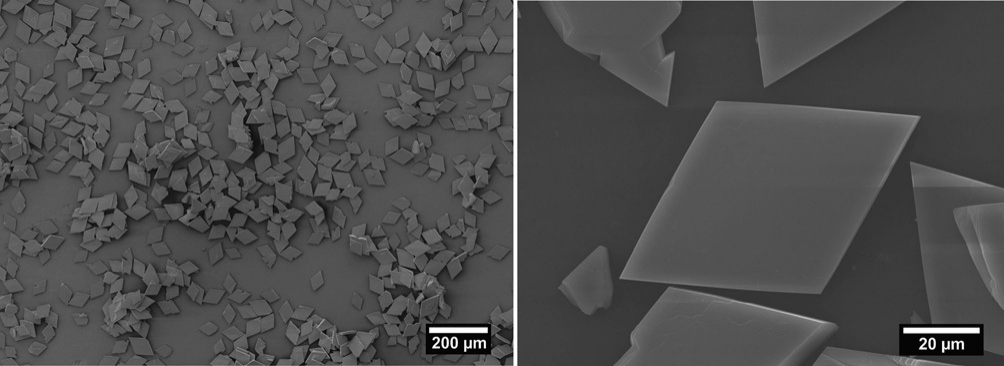
Representative SEM images of rhombic‐shaped Zr‐*bzpdc*‐MOF crystals used for the experiments.

**Figure 2:**
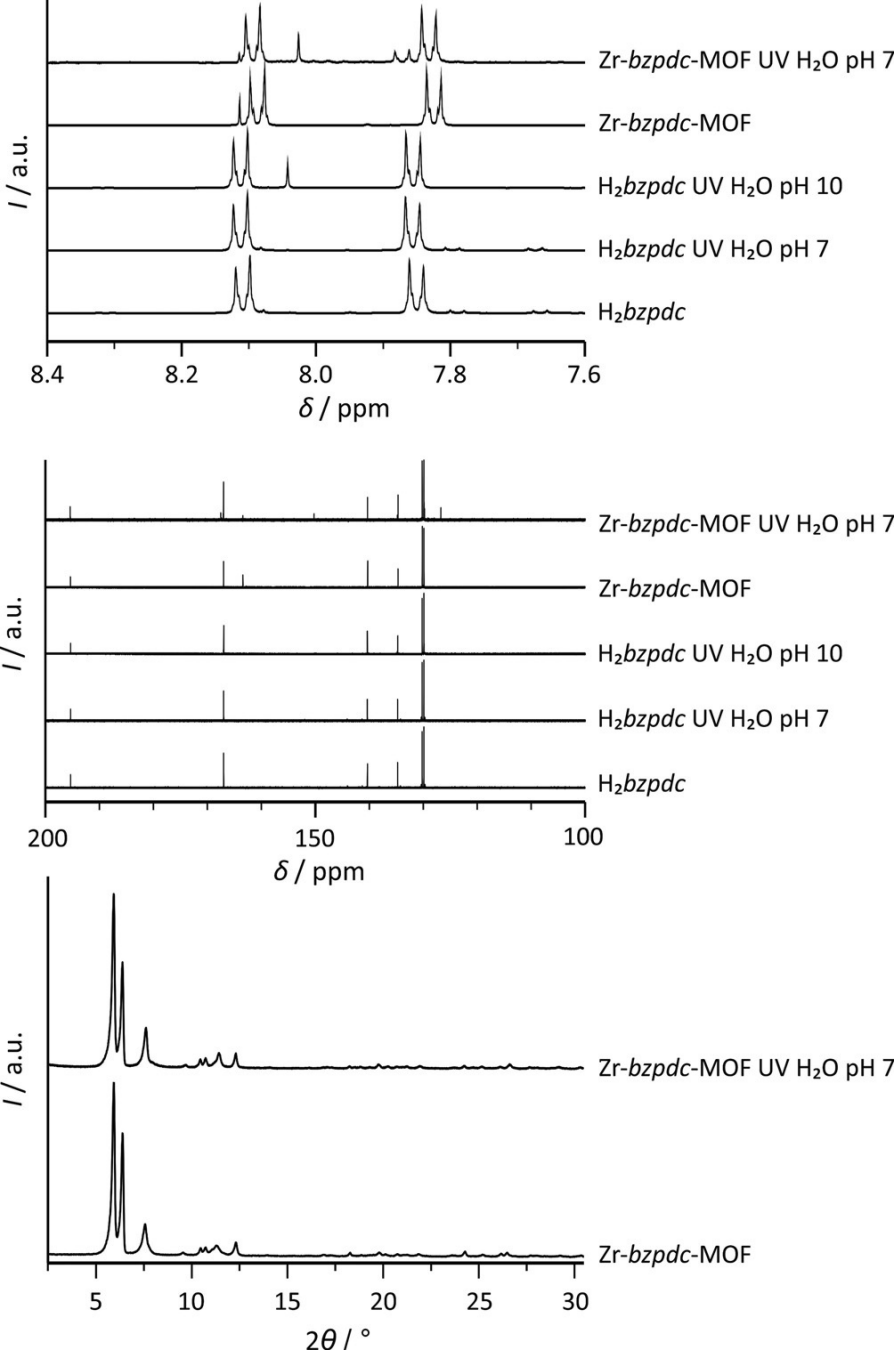
Characterization of the free diacid H_2_ *bzpdc* and of the Zr‐*bzpdc*‐MOF before and after the irradiation in water (irradiation duration of 72 hours). Selected ranges of ^1^H (top) and ^13^C (middle) NMR spectra, taken on dissolved (H_2_ *bzpdc*) and acid‐digested samples (Zr‐*bzpdc*‐MOF), respectively; bottom: PXRD patterns of the Zr‐*bzpdc*‐MOF before and after the irradiation in water.

**Figure 3:**
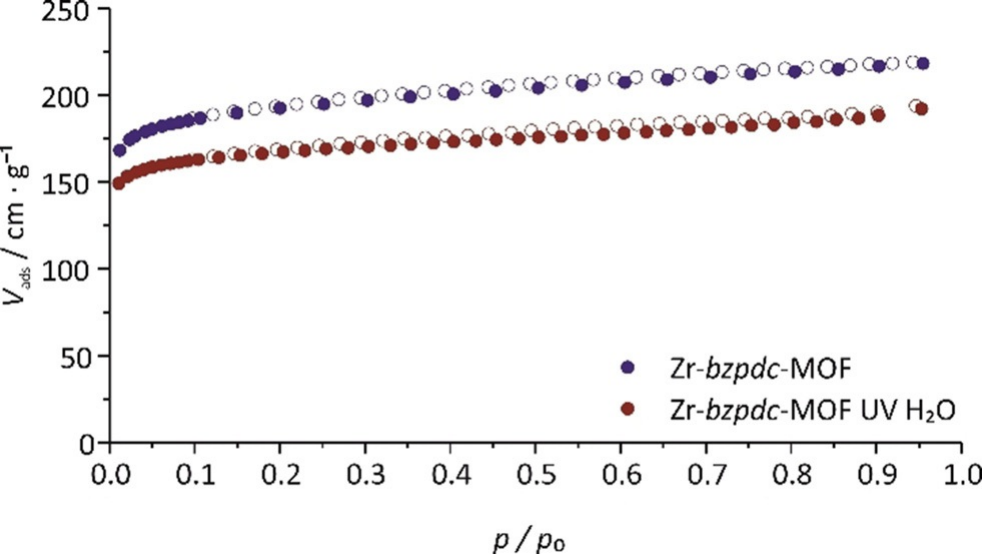
Nitrogen physisorption measurements at 77 K of the pristine Zr‐*bzpdc*‐MOF and of a sample irradiated in water.

The synthesis of Zr‐*bzpdc*‐MOF was adapted from our already reported synthesis route^24^ and leads to rhombic shaped crystals with edge length of about 80–100 μm, as shown in Figure 1. The material was extracted with acetone in a Soxhlet extractor for 24 hours, dried and kept under reduced pressure. Essential properties, like crystallinity and sorption behaviour, are shown in Figure 2 and Figure 3. With a BET area of about 680 m^2^ g^−1^ and a total pore volume of about 0.3 cm^3^ g^−1^, the porosity parameters calculated from the data shown are in very good agreement to the published values.^24^ In order to ensure reproducibility and consistency of our studies, the synthesis approach was scaled up to obtain in one batch a sufficient amount of Zr‐*bzpdc*‐MOF for all post‐synthetic modification reactions and the application of the various characterisation methods.

### General aspects of the photochemical reactions

For every post‐synthetic treatment, the sample was directly dispersed in the neat reactant and purged with argon before irradiation. Irradiated samples are designated by “UV”, followed by the reactant (and possibly other relevant conditions), throughout the paper. A suitable characterisation method to evaluate the PSM of the Zr‐*bzpdc*‐MOF is solution‐state NMR spectroscopy, performed on acid‐digested samples. Further characterisation methods applied are powder X‐ray diffraction and physisorption experiments.

**Scheme 2:**
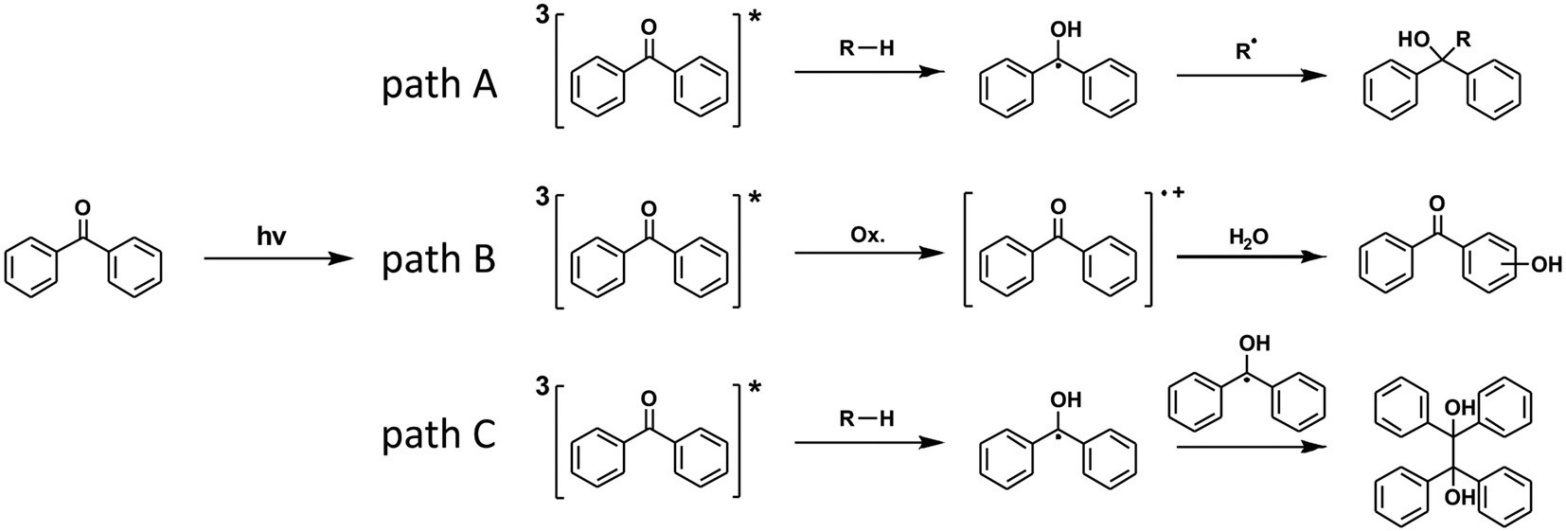
Possible reaction paths of a photoexcited benzophenone unit. Path A: Addition of R at the former keto carbon atom, formation of an alcohol. Path B: Addition of R at an aromatic ring, formation of an alcohol. Path C: Formation of benzopinacol.

In principle, photoexcited benzophenone can react in different ways,^31^ as depicted in Scheme 2. Formation of the benzophenone diradical can be followed by the abstraction of an H atom from a substance RH and subsequent addition of R to the former keto carbon atom (path A). However, a radical electron of the photoexcited benzophenone can also become delocalized over a neighbouring aromatic ring, as exemplarily shown in path B for the reaction with water (see below) which leads to a hydroxylation of the benzene ring (path B).^30^, ^34^ Both path A and path B could also occur with benzophenone moieties which are part of the framework. Another often observed reaction of photoexcited benzophenones is the formation of a benzopinacol via dimerisation (path C), typical for example for the reaction with 2‐propanol; a similar reaction should be possible with methanol.^35^ However, this does not appear to be possible in the MOF structure, due to the large distances between the keto groups (smallest distance of about 4.8 Å) and the geometric constraints of the framework.

### Photochemical reactions in water

We first studied photochemical reactions in water. The reactivity towards water is of special concern, because as part of the MOF framework, all the benzophenone moieties are exposed, and even air humidity might lead to a reaction under light exposure. Actually, there are only very few studies in the literature for the reaction of benzophenone moieties in aqueous media.^30^, ^31^, ^32^, ^33^ For the studies of the photochemical reactivity towards water, we used the Zr‐*bzpdc*‐MOF and, for comparison, the free linker molecule H_2_ *bzpdc* to obtain fundamental insights into possible differences in photoreactivity.

We investigated the photochemical reactivity of Zr‐*bzpdc*‐MOF in water at a pH of 7 and for comparison, of the free linker molecule H_2_ *bzpdc* in water of pH 7 and pH 10. The pH values were adjusted with sodium hydroxide and a pH meter. The MOF was of course present as a dispersion; the free diacid formed a dispersion at pH 7 and was dissolved at the higher pH value. The most obvious effect of the photochemical reaction is a colour change from colourless to dark yellow upon irradiation. This was observed for irradiated Zr‐*bzpdc*‐MOF and for H_2_ *bzpdc* under basic conditions. For H_2_ *bzpdc* irradiated in water at a neutral pH value, no colour change could be observed which can be ascribed to the insolubility of the free acid and the inaccessibility of the molecules.

More detailed results concerning the photochemical reaction can be obtained by NMR spectroscopic investigations on solutions. For this purpose, the MOF samples were digested in [D_6_]DMSO with hydrofluoric acid whereas the irradiated benzophenone dicarboxylic acid samples were dissolved in [D_6_]DMSO. The crucial regions of the NMR spectra of irradiated samples of H_2_ *bzpdc* and Zr‐*bzpdc*‐MOF are shown in Figure 2 (full spectra are shown in the Supporting Information, Section 1.1, Figures S1–S10).

PXRD patterns of the pristine Zr‐*bzpdc*‐MOF and the irradiated sample in water (Figure 2) show a comparable crystallinity and there are no indications for major changes in the crystal structure.

^1^H NMR spectra in Figure 2 show the characteristic multiplet of the aromatic hydrogen atoms of the benzophenone moieties. The additional signal at about 8.11 ppm for dissolved Zr‐*bzpdc*‐MOF samples belongs to the formic acid molecules which are an integral part of the MOF structure.

After irradiation of H_2_ *bzpdc* under basic conditions and Zr‐*bzpdc*‐MOF in water at pH 7 an additional proton signal appears at 8.03–8.04 ppm (Figure 2) in the ^1^H NMR spectra, indicating the formation of an *o*‐hydroxybenzophenone moiety. That the reaction occurs according to path B and not to path A (see Scheme 2) is supported by the ^13^C NMR spectra, where the signal at about 195 ppm, characteristic of the keto carbon atoms, remains mainly unchanged. The differences and the additional signals in the area of 160–170 ppm could be assigned to the aromatic carbon atoms that are affected by the hydroxylation. Based on the ^1^H NMR spectra, a comparison of the intensities of the additional signals to the signals of those of the other aromatic protons allows the estimation that about 70 % of the linker molecules have become hydroxylated in case of H_2_ *bzpdc* and about 50 % in case of Zr‐*bzpdc*‐MOF, respectively. For the MOF samples, physisorption measurements were performed (Figure 3).

The photochemical PSM affects the pore system of the MOF only to a minor degree, with only slightly decreased values for the BET area and pore volume (see Supporting Information, Section 1.2).

With the proposed reaction according to path B in Scheme 2, where hydroxylation occurs at the phenylene moieties and not at the keto carbon atom, the hybridisation at the central C atom remains *sp* ^2^ and, correspondingly, the general geometry of the linker does not change significantly. Thus, the general structure of the framework is not affected by the PSM reaction, which explains that only minor changes occur in the powder XRD pattern. Also, the addition of small OH groups does not significantly affect the pore system, so that also the physisorption properties after the reaction with water are similar.

The PSM of Zr‐*bzpdc*‐MOF with water leads to a post‐synthetic modification of the linker molecules while preserving its crystallinity and its porosity. It is apparently a suitable and very simple method to introduce hydroxy groups to the framework which should greatly increase the hydrophilicity with regard to the parent Zr‐*bzpdc*‐MOF.

### Photochemical reactions in methanol and ethanol

The photochemical potential of benzophenone groups to bind to molecules containing C−H bonds has been well known for many decades.^23^, ^34^, ^36^, ^37^, ^38^ The Zr‐*bzpdc*‐MOF possesses high chemical and good thermal stability, basic prerequisites for successful post‐synthetic reactions. According to its crystal structure, all benzophenone moieties present throughout the framework are accessible via the pore system; in addition, the rhombic‐shaped crystals present benzophenone groups on their predominant basal surfaces.^24^ The Zr‐*bzpdc*‐MOF exhibits moderate permanent porosity consisting of corrugated channels with a minimum free pore diameter of 6.5 Å (visualized in the Supporting Information, Section 1.3, Figure S13).

In initial studies, we had shown the general possibility to perform PSM reactions on Zr‐*bzpdc*‐MOF crystals. As reactants we had used polyethylene glycol and decane (and could in this way change the wetting behaviour of the MOF crystals)^24^ or EDOT (and could in this way prepare electrically conducting composites).^29^ However, in these reactions only the benzophenone groups on the outer surface had reacted. Clearly, the reactants mentioned cannot enter the pore system and therefore could not approach the inner keto groups.

**Figure 4:**
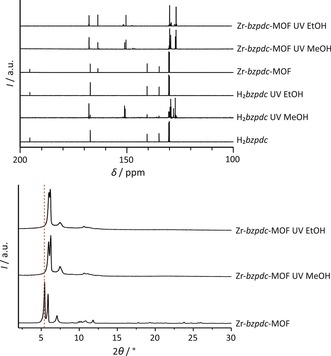
Characterisation of the free acid H_2_ *bzpdc* and of Zr‐*bzpdc*‐MOF samples (which were acid‐digested for NMR preparation) after irradiation in methanol and ethanol, respectively (duration of irradiation: 120 hours). Top: solution‐phase ^13^C NMR spectra; bottom: PXRD patterns (the position of the first reflection of the pristine Zr‐*bzpdc*‐MOF is marked with a dotted red line).

Therefore, we studied in depth the corresponding reactions with smaller C−H‐bond containing molecules, namely methanol and ethanol, again comparing the reactivity of the Zr‐*bzpdc*‐MOF and the free acid H_2_ *bzpdc*. For this purpose, the materials were dispersed in the corresponding alcohol and irradiated under argon atmosphere for 120 hours. H_2_ *bzpdc* itself is only slightly soluble in methanol and ethanol. However, during irradiation the free acid dissolved completely in methanol but still not in ethanol. Methanol and ethanol were removed under reduced pressure at room temperature. Afterwards, the samples were characterised via powder X‐ray diffraction and NMR spectroscopy (Figure 4).

Considering the reaction of the free acid in methanol, the ^13^C NMR spectrum of the product (Figure 4 top) shows that the signal of the keto carbon atom signal at about 195 ppm is missing, indicating a successful and complete reaction of methanol with H_2_ *bzpdc*. A detailed inspection of the ^1^H NMR spectra (see Supporting Information, Section 1.4, Figure S14) does not allow to assign the signals present to one specific product.

On the contrary, the irradiation of the free acid in ethanol does not lead to a reaction as all the ^13^C NMR signals of the original molecule are still present (see Supporting Information, Section 1.4, Figure S15). This can be explained by the insolubility of H_2_ *bzpdc* in ethanol so that there is no access to the keto groups occluded within the dispersed particles. Furthermore, the NMR spectra show that a possible presence of water in the alcohols does not notably influence the reaction of the keto groups with the alcohols.

**Scheme 3:**
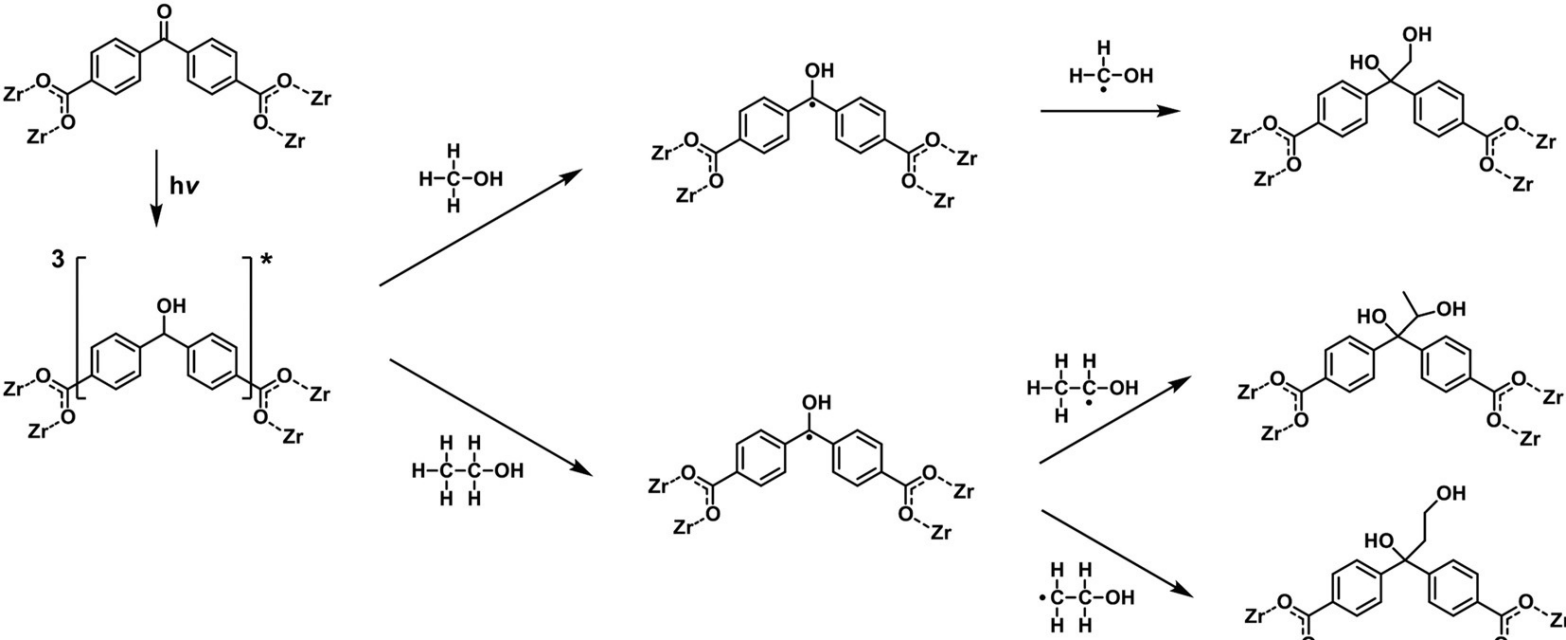
Reactions of a photoexcited benzophenone linker in Zr‐*bzpdc*‐MOF with methanol (top branch) and ethanol (bottom branch). Note that two different products are possible for the PSM with ethanol.

The Zr‐*bzpdc*‐MOF shows a successful and complete reaction after irradiation with both alcohols. The ^13^C NMR spectra in Figure 4 proof a practically complete conversion of the keto groups of the MOF after irradiation by the absence of the keto carbon signal in the corresponding ^13^C NMR spectra at about 195 ppm (Figure 4 top). This points to a conversion according to path A. The corresponding reactions are shown in Scheme 3. Note that the reaction with ethanol can lead to two different products depending on which of the two carbon atoms of the ethanol molecule becomes attached to the former keto carbon atom. In addition, the signal of the carbon atoms of the benzene rings in alpha position to the keto group experience an upfield shift from 140 ppm to about 150 ppm; this is a strong hint of the reduction of the keto group to an alcohol during the reaction with methanol or ethanol and of a concomitant change of the hybridisation of the keto carbon atom from *sp* ^2^ to *sp* ^3^. Also, the signals for the aliphatic carbon atoms of methanol and ethanol, respectively, can be observed in the corresponding ^13^C NMR spectra at 70–80 ppm (see Supporting Information, Section 1.4, Figure S16–19). Notwithstanding, as in the case of the reaction product of the free acid with methanol, the signals present cannot be assigned to a specific product.

Powder X‐ray diffraction patterns of the reacted MOFs are shown in Figure 4 (bottom). These indicate a structural change of the framework. This is in contrast to the PXRD patterns obtained from samples which had been reacted with water (see above) and with larger molecules like decane and polyethylene glycol^24^ as well as with 3,4‐ethylenedioxythiophene.^29^ In these cases, no differences in the PXRD pattern were observed with regard to the parent Zr‐*bzpdc*‐MOF which is taken as evidence that only surface‐standing keto groups had participated in the photochemical reaction. The changes in the PXRD patterns of the samples which were reacted with methanol or ethanol can be rationalised when it is assumed that the photochemical reaction has taken place throughout the MOF crystals. In the course of such reactions, (at least the major part of) the keto carbon atoms change their hybridisation from *sp* ^2^ to *sp* ^3^, leading to a framework with the same linkages and topology as the parent framework, but with different dimensions due to the change in linker geometry. However, due to the different possibilities for the reaction, where addition of the hydroxyalkyl group can occur at different sites of the benzophenone moiety and—in the case of ethanol—at different carbon atoms of the alcohol, these novel MOFs will not have a uniform structure which could be described by a single unit cell (see below).

These results lead to the hypothesis that the molecule size is a major factor in determining whether the post‐synthetic addition reaction takes place only at the outer surface of or throughout the crystals. Therefore, we carried out photochemical PSM reactions with primary linear alcohols of chain length C_1_ to C_8_ (methanol to octanol), including methanol and ethanol which were studied here in depth. Along with the size of the molecules, their polarity and hydrophilicity also change. Therefore, in order to also test the possible influence of polarity of the reactant, reactions with linear alkanes (butane to octane) were included in the study.

### Photochemical reactions with alcohols and alkanes of different chain lengths

To test whether the size and/or the polarity of reactant molecules is a major factor in determining the extent of the post‐synthetic addition (surface only/pervasive), we carried out photochemical PSM reactions of the Zr‐*bzpdc*‐MOF with linear alcohols of chain length C_1_ to C_8_ (methanol to octanol) as well as with linear alkanes (butane to octane). In the first part, we focus on the reactions with primary alcohols, thereby including the results described for the PSM reactions with methanol and ethanol in the preceding section.

**Figure 5:**
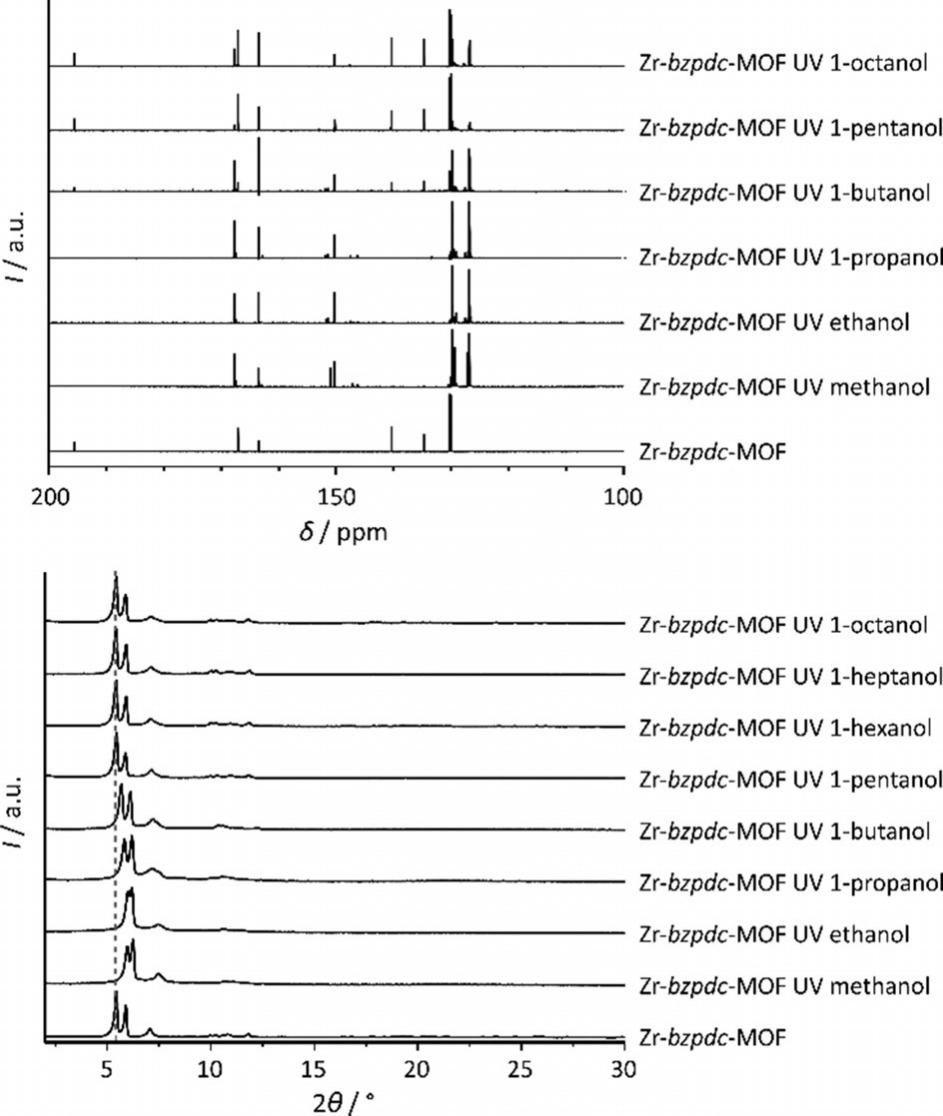
Characterisation of the pristine Zr‐*bzpdc*‐MOF and Zr‐*bzpdc*‐MOF samples after irradiation in different alcohols (duration of irradiation: 120 hours). Top: Solution‐phase ^13^C NMR spectra; samples were acid‐digested for NMR preparation. Bottom: PXRD patterns (the position of first reflection of Zr‐*bzpdc*‐MOF is marked with a dotted red line).

To investigate whether the PSM reaction with a certain alcohol was successful, the samples were characterised via powder X‐ray diffraction and NMR spectra of the acid‐digested reaction products. PXRD patterns are shown in Figure 5 (bottom) and selected ranges of the obtained ^13^C NMR spectra are displayed in Figure 5 (top) (full NMR spectra are provided in the Supporting Information, Section 1.4, Figure S14–S28). The intensity of the signal of the keto carbon atom is most informative.

After the reaction carried out in 1‐propanol, no keto carbon signal is observed anymore; instead, signals for aliphatic carbon atoms at around 70–80 ppm are found. This result is comparable to the situation with methanol and ethanol. On the contrary, in the case of the reaction products obtained with 1‐pentanol and also 1‐octanol, the keto carbon signal is still present, indicating that the keto groups have not reacted or have done so only to a very small extent. After the irradiation in 1‐butanol, the keto carbon signal in the ^13^C NMR spectrum of the reaction product is very small, indicating that the reaction with this alcohol was also nearly complete. In fact, when the Zr‐*bzpdc*‐MOF is stirred for 24 hours in butanol before irradiation, the following photochemical reaction is complete, as judged from the ^13^C NMR spectrum of the acid‐digested product (see Supporting Information Figure S24) where no keto carbon signal can be observed anymore. Therefore, it appears that in the reaction with butanol the diffusion of the reactant molecule through the narrow and corrugated channels of the host structure is an additional factor influencing the extent of the reaction. Thus, the PSM with 1‐butanol appears to be a borderline case: Irradiation in smaller alcohols lead to a full reaction of the keto groups whereas larger alcohol molecules probably only react at the keto groups located at the outer surface of the MOF crystals.

This delineation also becomes apparent in the powder X‐ray diffraction patterns (Figure 5, bottom). The PXRD patterns of the samples that were irradiated with long‐chain alcohols (pentanol to octanol) show no changes in comparison to that measured on the pristine Zr‐*bzpdc*‐MOF. In contrast, for those samples which were post‐synthetically modified with small alcohols (methanol to butanol), changes can be observed: the first reflections are shifted to slightly higher 2*Θ* values, the reflection pattern at larger 2*Θ* values changes, and in general a minor decrease in crystallinity is indicated by weaker and broader reflections in this range.

**Figure 6:**
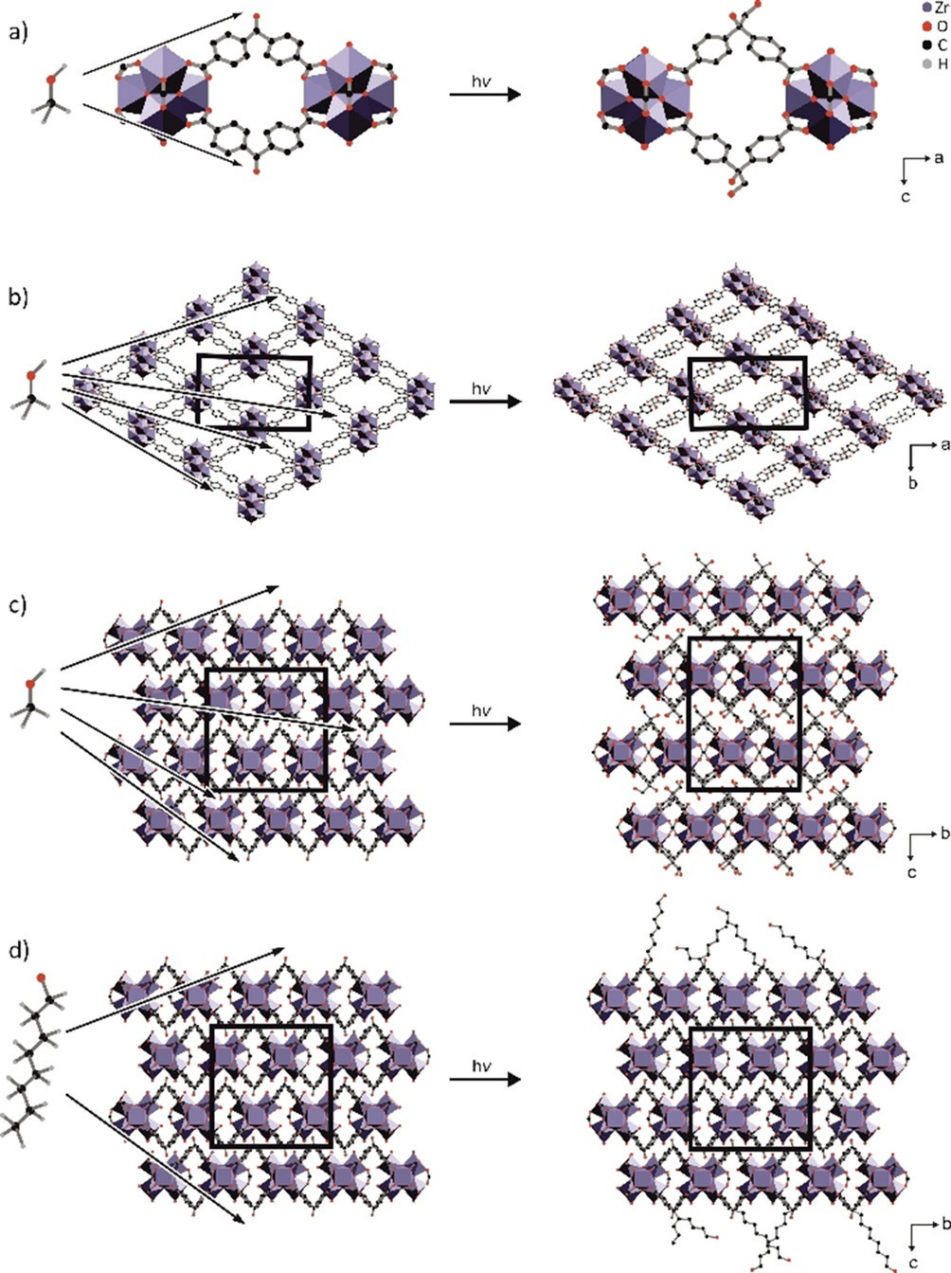
Schematic representation of different reactions occurring during irradiation of the Zr‐*bzpdc*‐MOF, depending upon the chain lengths of C−H bond‐containing molecules (alcohols are shown here as examples). a)–c) Small alcohol molecules (methanol is shown here as example) can diffuse through the whole pore system and the PSM reaction can occur with every photoexcited benzophenone group leading to considerable structural changes: a) at the keto carbon atom, the hybridisation changes from *sp* ^2^ to *sp* ^3^; b) the change in bond angles leads to a shrinkage of the unit cell in the *a*‐*b* plane; c) the additional space required by the hydroxymethylene residues leads to an elongation along the c axis. d) Long‐chain alcohols (octanol is shown here as an illustrative example) cannot penetrate deeply into the framework; only benzophenone moieties at or near to the surface react, the structure and the unit cell within the interior of the crystals is not affected.

The shift of the two first, most intense reflections (110 and 200) to higher 2*Θ* values can be rationalised by the changes in the linker geometry. Each linker molecule connects two IBUs in the structure of the Zr‐*bzpdc*‐MOF. In course of the photochemical PSM reaction, the hybridization of the initial keto carbon atom changes from *sp* ^2^ to *sp* ^3^ by the photochemical reaction (Figure 6 a). Correspondingly, the bond angles at this carbon atom change from 120° to 109.5° which in turn should result in a shrinkage of the distance between two IBUs on certain lattice planes, provided that the topology of the framework remains the same and that every keto group (or most of them) is affected (Figure 6 a). Concomitantly, changes occur in the unit cell dimensions (see Supporting Information, Section 1.6, Table S2).

This is shown in Figure 6 a–c for the product of the reaction with methanol. Note that this is the only case where a homogenous structure of the product can be expected (when all keto groups have reacted) and where a uniform unit cell can be determined. In all other cases, different C atoms of the alcohol may become connected to the keto C atom (compare the case of ethanol depicted in Scheme 3), leading to different situations at different transformed keto C atoms. Therefore, we carried out simulations of the structure resulting after the PSM reaction with methanol.

The *a* and especially the *b* axis shrink (Figure 6 b), explaining the shift of the first two reflections to higher 2*Θ* values (Figure 5 bottom). The hydroxymethylene residues introduced at the former keto carbon atom through the PSM require additional space between the layers of the structure, thereby leading to an expansion of the structure along the *c* axis (Figure 6 c). On the contrary, the bulk structure is not affected when the reaction occurs only at the surface, as illustrated in Figure 6 d.

**Figure 7:**
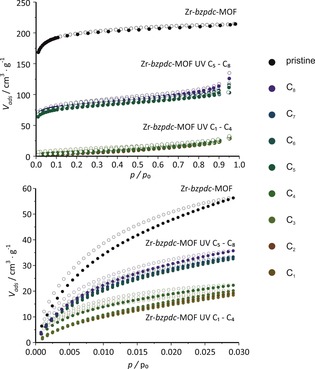
Physisorption isotherms of the pristine Zr‐*bzpdc*‐MOF and of Zr‐*bzpdc*‐MOF samples irradiated in primary alcohols with different chain lengths (C_1_–C_8_ denotes methanol to 1‐octanol). Top: Physisorption isotherms obtained with N_2_@77 K; bottom: physisorption isotherms obtained with CO_2_@273 K.

The irradiated samples were further investigated by physisorption measurements with nitrogen at 77 K and carbon dioxide at 273 K. A compilation of the resulting isotherms of the N_2_@77 K measurements is shown in Figure 7 (as many of the isotherms closely overlap, the individual sorption curves are shown in the Supporting Information, Section 1.2, Figure S11; values for BET area and pore volume derived from the nitrogen sorption isotherms are given in the Supporting Information, Section 1.2, Table S1). In comparison to the pristine Zr‐*bzpdc*‐MOF with a BET area of 680 m^2^ g^−1^, the samples modified with long‐chain alcohols (1‐pentanol to 1‐octanol) only show about half of this value (ca. 350 m^2^ g^−1^). Similar values for the nitrogen BET area were observed in our former studies after the irradiation in PEG (250 m^2^ g^−1^),^24^ decane (350 m^2^ g^−1^)^24^ and EDOT (520–630 m^2^ g^−1^).^29^ The decreased amounts of gas volume adsorbed by these samples indicate a partial pore blocking of the pore systems due to PSM occurring only at the outer surface with long chain alcohols. When the PSM occurs not only at the surface, but throughout the crystals, as is the case with the alcohols methanol up to butanol, the porosity is very low. From the flat N_2_ isotherms depicted in Figure 7, BET areas of about 20 m^2^ g^−1^ are determined, that is, nitrogen molecules cannot enter anymore the former pore system.

Whereas the nitrogen physisorption isotherms of the samples treated post‐synthetically with methanol, ethanol, propanol and butanol thus indicate the formation of essentially nonporous products, this statement is negated by physisorption experiments with carbon dioxide at 273 K. Because of the high temperature and the high relative pressures (*p*/*p*_0_≈3×10^−2^) for this experiment, diffusion is much faster and therefore it is possible to resolve ultra‐small micropores (below 0.7 nm). Although the general trend in adsorbed volume of carbon dioxide is similar as for nitrogen (Figure 7 bottom), the samples treated post‐synthetically with short‐chain alcohols still exhibit a moderate free pore volume which is not measurable with nitrogen at 77 K, possibly due to diffusion hindrance. Based on these results, the pores of the samples should be below ca. 4 Å diameter. When, with alcohols with more than four carbon atoms the PSM occurs only at or near the surface, a larger accessible porosity results. Like in the case of nitrogen sorption, this porosity is considerably smaller than for the pristine Zr‐*bzpdc*‐MOF.

**Figure 8:**
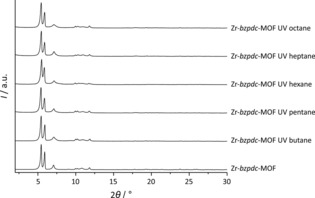
PXRD patterns of pristine Zr‐*bzpdc*‐MOF and of Zr‐*bzpdc*‐MOF samples irradiated in different alkanes.

A dependence of the amenability of the Zr‐*bzpdc*‐MOF to the photochemical modification on the chain lengths of the alcohols is thus clearly apparent. Although judging from their largest dimension (diameter of the methylene chain of the alcohols: 4.8 Å), all alcohol molecules should be able to enter the pore system of the Zr‐*bzpdc*‐MOF (minimum free pore diameter: 6.5 Å), the corrugated channel structure could strongly impede the diffusion of longer‐chain alcohols (C_5_ to C_8_) into the interior of the crystal whereas small‐chain alcohols (C_1_ to C_4_) can diffuse through the whole pore system (although the diffusion of butanol takes considerable time, see above). Another possibly important aspect is that with the chain length of the alcohols, their hydrophobic character increases, which could lead to a decreased diffusion into the pore structure of the rather hydrophilic Zr‐*bzpdc*‐MOF.^24^ Therefore, we furthermore tested the PSM reaction with more hydrophobic alkanes, starting with butane and increasing the chain length up to octane. For butane, the reaction was carried out in the liquid phase in a closed ampoule; reactions with even smaller alkanes were precluded due to the high vapour pressures of methane, ethane and propane at room temperature. When we acid‐digested the samples to measure NMR spectra, insoluble residues were still present after the treatment. Although we cannot exclude that part of the organic substances liberated by the digestion of the MOF may have become adsorbed on the solid, we have measured NMR spectra of the supernatants. The results obtained are in line with reactions which take place (nearly) exclusively at the surface (see Supporting Information, Section 1.5, Figure S29 and S30). The PXRDs pattern of the samples after the PSM reaction are compared to that of Zr‐*bzpdc*‐MOF in Figure 8.

**Figure 9:**
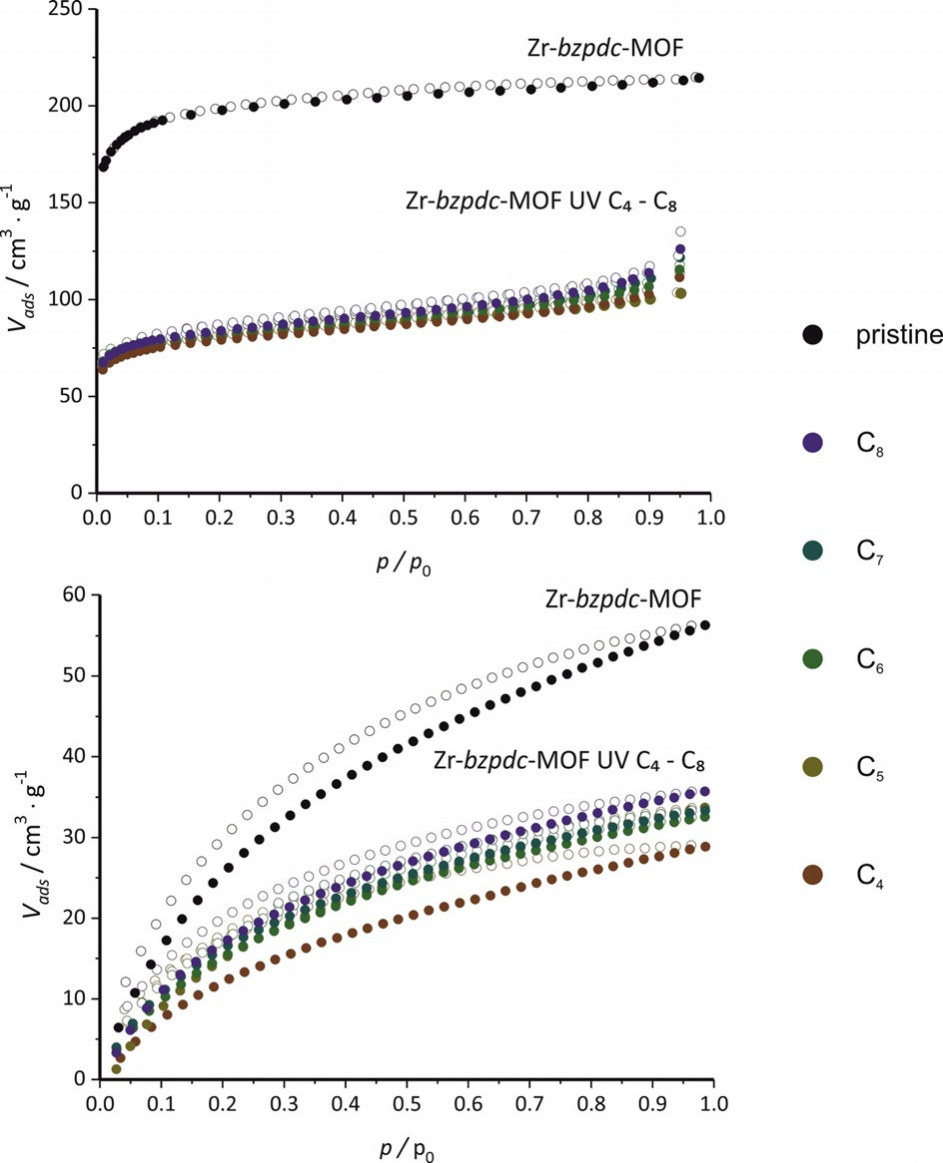
Physisorption isotherms of the pristine Zr‐*bzpdc*‐MOF and of Zr‐*bzpdc*‐MOF samples irradiated in linear alkanes with different chain lengths (C_4_–C_8_ denotes *n*‐butane to *n*‐octane). Top: Physisorption isotherms obtained with N_2_@77 K; bottom: physisorption isotherms obtained with CO_2_@273 K.

In case of the samples post‐synthetically modified with alkanes, no changes can be observed in the X‐ray diffraction patterns. Even for butane, the reflection positions and intensities are comparable to those of Zr‐*bzpdc*‐MOF. The PXRD results are corroborated by physisorption measurements, carried out with nitrogen at 77 K and carbon dioxide at 273 K, shown in Figure 9 (as many of the isotherms closely overlap, the individual sorption curves are shown in the Supporting Information, Section 1.2, Figure S12; values for BET area and pore volume derived from the nitrogen sorption isotherms are given in the Supporting Information, Section 1.2, Table S1).

Nitrogen physisorption isotherms show a considerable decrease in adsorbed volume for all post‐synthetically modified samples. The pore volumes and BET areas are in the range of about 350 m^2^ g^−1^, comparable to the values obtained on samples after reaction with long chain alcohols. A similar conclusion can also be drawn from the carbon dioxide isotherms.

The results show that also with butane—and in contrast to butanol—the PSM occurs only at the outer surface of the crystals, that is, no complete pervasion of the crystals occurs. This is a hint that in addition to molecular size also the polarity of the molecule may be important for the penetration of molecules into the framework and, accordingly, for the extent to which PSM occurs.

## Conclusions

The possibility for photochemical post‐synthetic modification of the recently introduced Zr‐*bzpdc*‐MOF is a special characteristic of this porous coordination polymer, as it can be carried out directly after synthesis and work‐up and does not need any further preparations. We have already shown that the photochemical reactivity of the benzophenone linker can be employed to adapt the surface properties of the MOF crystals^24^ and to generate interesting composite materials by direct grafting‐from polymerization reactions.^29^ Therefore, it is worthwhile to further our understanding of this reaction. We do this here by elucidating the question with which reactants a complete PSM extending throughout the crystal occurs and with which molecules the reaction is restricted to the surface. In this respect, we have shown that with small alcohol molecules (C_1_ to C_4_ alcohols), the PSM pervades the whole crystals whereas alcohol molecules with longer aliphatic chains (C_5_ to C_8_ alcohols) react only at the outer surface. Interestingly, in the borderline case *n*‐butanol, diffusion hindrance is possibly important. The polarity of the molecule probably also plays a role, as with the C_4_ alkane butane, the reaction occurs only at the outer surface.

Retrospectively, it is of interest to note the differences between the products of the PSM obtained after irradiation in PEG and decane which we had described in our original paper on the Zr‐*bzpdc*‐MOF.^24^ According to PXRD, the reactions only occurred at the outer surface. After the PSM in decane, nitrogen physisorption measurements reveal a BET area of 350 m^2^ g^−1^,^24^ agreeing very well the values obtained after PSM in C_5_ to C_8_ alcohols and C_4_ to C_8_ alkanes. With PEG, however, a value of only 250 m^2^ g^−1^ is obtained. This indicates that the very hydrophilic PEG molecules might have entered the pore system to some degree before they were restrained from further diffusion by their polymeric nature, and thus substantiates the idea that polarity of the reagent is an important factor in the selection between reactants which can react throughout the pore system and those which only react at the surface.

The concept of selective functionalisation of a MOF—either only at the surface or throughout the whole crystals—is of general interest for various applications like gas separation, optics, electronics or for the generation of composite materials. In case of the Zr‐*bzpdc*‐MOF, this can be accomplished photochemically directly after synthesis and work‐up. The selectivity of the functionalisation is closely coupled to the crystal structure of this MOF, namely on the one hand the exposure of reactive benzophenone moieties at the surface, on the other hand the peculiarities of the pore system, namely rather small apertures and corrugated channels as well as high hydrophilicity. In another *bzpdc*‐based MOF, namely CAU‐8 (Al‐*bzpdc*‐MOF), photochemical PSM reactions are also possible, but these always occur throughout the pore system.^27^, ^28^ In this respect, the synthesis of *bzpdc*‐based MOFs with other metals appears interesting, perhaps offering further selectivities with which the attractive reactivity of the benzophenone‐containing linker can be exploited.

## Experimental Section

**Materials**: Zirconium(IV)‐oxychloride octahydrate, formic acid, *N*,*N*‐dimethylformamide (DMF), *n*‐butane were purchased from Linde Group, pentane, hexane, heptane, octane, methanol, ethanol, 1‐butanol, 1‐pentanol, 1‐hexanol, 1‐heptanol, 1‐octanol were purchased from Merck Munich and benzophenone‐4,4′‐dicarboxylic acid from abcr chemicals Karlsruhe. All chemicals were used without further purification.

**Instrumentation**: Powder X‐ray diffraction (PXRD) measurements were performed at room temperature using a STOE STADI‐P transmission diffractometer equipped with curved Ge(111) monochromator with Cu_Kα1_ radiation (*λ*=1.540594 Å) and a linear position‐sensitive detector. The samples were fixed between X‐ray amorphous foils. Scanning electron microscopy was carried out with a JEOL JSM‐6700F (2 keV). The samples were prepared by the use of ethanolic dispersions dried on a graphite pad under reduced pressure. The resulting images were edited with the software ImageJ 1.49v. Liquid‐phase ^1^H and ^13^C NMR spectra were measured on a Bruker instrument at 400 MHz and were analysed with ACD NMR Processor 12. Sorption isotherms were measured at an Autosorb 1 (Ar) and Autosorb 3 (N_2_, CO_2_) instrument from Quantachrome and were evaluated with the software ASiQwin 2.0 (Quantachrome). Measurements were performed with nitrogen at 77 K and with carbon dioxide at 273 K. From nitrogen sorption isotherms, BET areas of the microporous samples were determined with the micropore BET assistant of the accompanying software and total pore volumes were determined at a relative pressure value of about 0.95. UV irradiation experiments were performed with a high power UV LED Spot epiSPOT by Laser 2000 with a wavelength of 365 nm and 176 mW optical power.

**Synthesis of (Zr_6_O_4_(OH)_6_(HCO_2_)_2_(*bzpdc*)_4_)**: For reproducibility and consistency, all post‐synthetic modification reactions were carried out on Zr‐*bzpdc*‐MOF single crystals synthesized in a one‐batch‐serves‐all approach by a route slightly modified from the already published recipe.^24^ Solvothermal synthesis of Zr‐*bzpdc*‐MOF was carried out in Teflon‐sealed screw cap glass vessels using a modulation approach.^25^, ^39^ Favourable conditions to obtain single crystal samples without impurities are as follows: 3.22 g (10 mmol) ZrOCl_2_⋅8 H_2_O were dissolved in 200 mL DMF. After adding 56.59 mL formic acid (150 molar equivalents (eq) based on amount of Zr^4+^) and 5.40 g (20 mmol) H_2_ *bzpdc* the clear solution was transferred in a 500 mL Teflon‐sealed glass vessel and was kept at 120 °C for 2 weeks in a circulating air oven. After the reaction, the solution was cooled down to room temperature and the resulting solid was centrifuged and washed once with 100 mL of DMF and two times with 50 mL acetone and then dried under reduced pressure. For further investigations the sample was activated via Soxhlet extraction with acetone for 24 hours in the dark. The white powder was kept under reduced pressure until further usage.

**Post‐synthetic modification**: The photochemical reactions were performed using a UV‐LED with a wavelength of 365 nm and a radiant flux of about 100 mW cm^−2^. A MOF sample of about 100 mg was placed in a quartz cuvette and ca. 3 mL of the neat reactant were added. The reaction batch was flushed for 30 minutes with and kept under argon while irradiating the samples under stirring. The irradiation time was set to 120 hours for alkanes and alcohols. For investigations in aqueous media the irradiation times were 72 hours. After irradiation, the samples were extracted for 24 hours with methanol (Soxhlet extractor) and dried under reduced pressure. For the post‐synthetic modification with butane, the MOF was sealed with 5 mL liquid butane in a quartz vial with a stir bar. After irradiation for 120 hours under stirring, unbound molecules were removed under reduced pressure. This sample was used for further investigations without Soxhlet extraction.

**Preparation for solution‐phase NMR spectroscopy**: For liquid‐state NMR experiments, 50 mg of the MOF were dispersed in [D_6_]DMSO. Under vigorous stirring 20 μL aqueous HF (40 %) were added and stirred for 18 hours. After complete dissolution of the Zr‐*bzpdc*‐MOF, an excess of CaCl_2_ was added to the clear solution. The supernatant was used for ^1^H and ^13^C BB NMR investigations. The spectra were analyzed with ACD/NMR Processor Academic Edition v12.01.

**Structural modelling**: To investigate structural changes of the MOF by the PSM with methanol, the program suit Materials Studio from Dassault Système BIOVIA was used. First, the crystal structure of pristine Zr‐*bzpdc*‐MOF was subjected to an energy minimisation employing the UFF (Universal Force Field) within the Forcite program.^40^, ^41^, ^42^ The minimisation employed the Ewald summation method for electrostatic interactions. For this kind of interaction as well as for the atom‐based van der Waals interactions the cut off was set to 15 Å. In the first step of the minimisation procedure, only atom coordinates were relaxed while the cell parameters were retained. In the second step, both the unit cell parameters and the atoms were allowed to relax. In order to find the global minimum, this structure was employed for a Quenched Dynamics procedure leading to 10 000 energy‐minimised structures. The parameters are given in Table 1.

**Table 1:** Parameters for the Quenched Dynamics employed to the model structures.

Parameter	Value
Ensemble	*NPT*
Temperature	1000 K
Thermo‐ and barostat	Berendsen
Decay constant	0.1 ps
Time step	1 fs
Total simulation time	20 ns
Frame output every	2000 steps
Final frames	10 000

Our model for the sample after PSM with methanol is based on the reaction according to path A. The *sp* ^2^ keto carbon atoms of the benzophenone units were transformed to *sp* ^3^ C atoms. Instead of the double‐bonded oxygen atoms, hydroxy groups and hydroxymethylene groups were placed at these carbon atoms. By doing so, this carbon atom is connected to the corresponding alkyl residue. This structural model then underwent similar procedures as described above for the pristine Zr‐*bzpdc*‐MOF.

## Conflict of interest

The authors declare no conflict of interest.
